# Modification of Superoxide Dismutase 1 (SOD1) Properties by a GFP Tag – Implications for Research into Amyotrophic Lateral Sclerosis (ALS)

**DOI:** 10.1371/journal.pone.0009541

**Published:** 2010-03-08

**Authors:** James C. Stevens, Ruth Chia, William T. Hendriks, Virginie Bros-Facer, Jan van Minnen, Joanne E. Martin, Graham S. Jackson, Linda Greensmith, Giampietro Schiavo, Elizabeth M. C. Fisher

**Affiliations:** 1 Department of Neurodegenerative Disease, UCL Institute of Neurology, London, United Kingdom; 2 Center for Regenerative Medicine, Massachusetts General Hospital, Harvard Medical School, Boston, Massachusetts, United States of America; 3 Sobell Department of Motor Science and Movement Disorders, UCL Institute of Neurology, London, United Kingdom; 4 Department of Cell Biology and Anatomy, Hotchkiss Brain Institute, University of Calgary, Calgary, Canada; 5 ICMS Pathology Group, Queen Mary University of London, London, United Kingdom; 6 MRC Prion Unit, UCL Institute of Neurology, London, United Kingdom; 7 Molecular Neuropathobiology Laboratory, Cancer Research UK, London, United Kingdom; National Institutes of Health, United States of America

## Abstract

**Background:**

Since the discovery that mutations in the enzyme SOD1 are causative in human amyotrophic lateral sclerosis (ALS), many strategies have been employed to elucidate the toxic properties of this ubiquitously expressed mutant protein, including the generation of GFP-SOD1 chimaeric proteins for studies in protein localization by direct visualization using fluorescence microscopy. However, little is known about the biochemical and physical properties of these chimaeric proteins, and whether they behave similarly to their untagged SOD1 counterparts.

**Methodology/Principal Findings:**

Here we compare the physicochemical properties of SOD1 and the effects of GFP-tagging on its intracellular behaviour. Immunostaining demonstrated that SOD1 alone and GFP-SOD1 have an indistinguishable intracellular distribution in PC12 cells. Cultured primary motor neurons expressing GFP or GFP-SOD1 showed identical patterns of cytoplasmic expression and of movement within the axon. However, GFP tagging of SOD1 was found to alter some of the intrinsic properties of SOD1, including stability and specific activity. Evaluation of wildtype and mutant SOD1, tagged at either the N- or C-terminus with GFP, in PC12 cells demonstrated that some chimaeric proteins were degraded to the individual proteins, SOD1 and GFP.

**Conclusions/Significance:**

Our findings indicate that most, but not all, properties of SOD1 remain the same with a GFP tag.

## Introduction

Amyotrophic lateral sclerosis (ALS) is a neurodegenerative disease affecting motor neurons in the motor cortex, brain stem and spinal cord. The first reports that mutations in superoxide dismutase 1 (*SOD1*) were causative for amyotrophic lateral sclerosis were published in 1993 [1], [2], and since then, over a hundred and forty mutations have been found [3]. Familial cases (fALS) account for 10–20% of all ALS, and up to 20% of fALS and ∼2% of sporadic ALS are caused by mutations in *SOD1*. However, although mutant SOD1 is now recognized to be the toxic moiety responsible for the selective death of motor neurons in SOD1-mediated ALS, the mechanism by which this protein exerts its toxicity is still not understood, and considerable effort is underway worldwide to analyse both the wildtype and mutant protein and to determine what causes death of motor neurons.

Analysis of SOD1 includes determining its subcellular localisation. Crapo and colleagues found wildtype SOD1 was localized to both the cytosol and nucleus of human cells, consistent with it being a soluble cytosolic protein; they also found that peroxisomes label less densely for SOD1 than cytoplasm [4]. Pardo et al. described intense SOD1 immunoreactivity in motor neurons, interneurons and the substantia gelatinosa of the mouse spinal cord [5]. The majority of this immunoreactivity appeared to be free within the cytoplasm although they describe a fraction associated with membranous organelles which they assumed to be peroxisomes. Recently, fluorescently tagging wildtype and mutant SOD1 with green fluorescent protein (GFP) or yellow fluorescent protein (YFP), to examine its intracellular localization, has become a well used method to explore possible novel mechanisms by which mutant SOD1 may exert its toxic effects. Examples of possible pathogenic mechanisms are the selective localization of mutant SOD1 to mitochondria resulting in caspase activation [6], formation of cytotoxic SOD1 aggregates which may act as a sink in sequestering other cellular proteins and so perturbing their normal functions [7], [8], [9], [10] and the selective inhibition of anterograde fast axonal transport causing a cargo specific alteration in axonal trafficking resulting in depletion of mitochondria in axons [11]. In these reports tagging GFP or YFP to SOD1 to allowed direct visualization of its localisation in cells. However, little is known about the biochemical consequences of GFP-tagging on SOD1. Considering that GFP, at 263 amino acids (molecular weight 27–30 kDa depending on the sequence) is considerably larger than monomeric mature SOD1 (153 amino acids, molecular weight 16 kDa per monomer) it is possible the presence of a GFP-tag on SOD1 may affect its function, warranting caution in interpreting results of experiments using these chimeric proteins. (We note that from cDNA sequence, human SOD1 contains 154 amino acids, but the start methionine is cleaved post-translationally, followed by N-acetylation of the first alanine, therefore making the number of actual amino acids in SOD1, 153.)

Here, we demonstrate that while GFP tagging of SOD1 does not appear to alter its cellular localization (at least in PC12 cells), it does alter some physicochemical properties of SOD1, suggesting that results from studies using GFP-tagged SOD1 should be interpreted with this in mind.

## Results

### Reduction in SOD1 Enzyme Activity when Tagged with GFP

**Figure 1:**
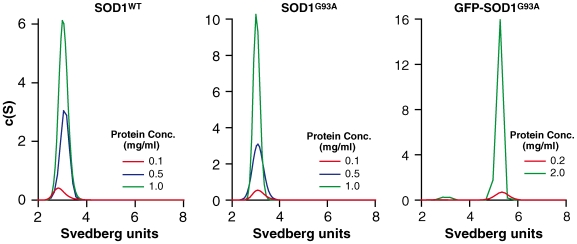
Dimeric stability of SOD1: wildtype, SOD1^G93A^ and GFP-SOD1^G93A^. Sedimentation velocity profile of SOD1 proteins. Proteins were analysed at three concentrations, the analysis included all 200 scans acquired during the experimental runs, which were analysed using the Sedfit version 9.2 based on the continuous size distribution model described in [25], [26]. The larger the *s*-values, the larger the size of the sedimenting protein species. Non-GFP tagged SOD1 dimers sedimented between 2.8 and 3.1S. GFP-SOD1^G93A^ sedimented at 5.3S, which was estimated to be approximately 88 kDa corresponding to the size of dimeric GFP-SOD1^G93A^. Wildtype SOD1, SOD1^G93A^ and GFP-SOD1^G93A^ existed almost exclusively as SOD1 dimers.

We characterized wildtype human SOD1 (SOD1^WT^), and mutant human SOD1 (SOD1^G93A^) with the G93A amino acid change, one of the most studied mutations of SOD1 both in vitro and in animal models of ALS [12]. We compared these two untagged proteins to SOD1^G93A^ tagged at its amino-terminus with GFP, written here as ‘GFP-SOD1^G93A^‘. Enzymatic activity was determined for the three proteins (assessing three separate samples, values expressed as the mean activity±SEM): while SOD1^WT^ (3705±331U/mg) and SOD1^G93A^ (3720±28U/mg) had comparable specific activities, the specific activity of GFP-SOD1^G93A^ was reduced by 65% (1304±101U/mg). Loss of activity was unlikely due to monomerization of GFP-SOD1^G93A^ because analytical ultracentrifugation (AUC) revealed GFP-SOD1^G93A^ existed as a stable dimer, with comparable dimeric stability to SOD1^WT^ and SOD1^G93A^ (Figure 1). Non-GFP tagged SOD1 dimers sedimented between 2.8–3.1S. This range of s-values was automatically calculated, using the Sedfit software, by converting the c(s) distribution into a c(M) distribution which will give the molar mass estimate based on the best-fit frictional ratio, to correspond to SOD1 with molecular weights of 31–35kDa. Wildtype SOD1, SOD1^G93A^ and GFP-SOD1^G93A^ existed almost exclusively as SOD1 dimers. GFP-SOD1^G93A^ sedimented at 5.3S, which was estimated to be ∼88kDa corresponding to the size of dimeric GFP-SOD1^G93A^.

Thus we saw a marked reduction in activity of GFP-SOD1^G93A^ compared to wildtype SOD1 and untagged SOD1^G93A^, and yet no difference in their dimerization states although dimerization is an essential post-translational modification for SOD1 to be enzymatically active.

### Higher Thermal Protein Stability of GFP-SOD1^G93A^ in the Metallated State, with an Increase of Stability when Demetallated

In the metallated form, SOD1^WT^ and SOD1^G93A^ had higher apparent Tm values than the demetallated form. The protein stability of SOD1 is not greatly influenced by protein concentration as there was only a 1–2°C change in the apparent Tm of proteins at 33µM versus 11µM, as shown in Table 1. Demetallation caused a significant reduction in protein stability of SOD1^WT^ and SOD1^G93A^ by ∼20°C. As expected, SOD1^G93A^ had a slight, but significant, decrease in the apparent Tm compared to SOD1^WT^, and this was consistent when Cu and Zn were removed with excess EDTA. Metallated GFP-SOD1^G93A^ had an apparent Tm value which was 5–6°C higher than the apparent Tm of metallated SOD1^WT^. Unexpectedly, demetallation of GFP-SOD1^G93A^ caused an increase in the apparent Tm which was an unusual effect compared to the demetallation effect seen in the non-GFP tagged SOD1 proteins (decreased apparent Tm corresponds to decreased protein stability).

**Table 1:** Thermal stability of metallated and demetallated wildtype SOD1, SOD1^G93A^ and GFP-SOD1^G93A^ proteins.

SOD1 variant	Apparent Tm (°C) (Metallated SOD1)	Apparent Tm (°C) (Demetalled SOD1)	p-value (two-tailed Student t-test for the paired condition)
**33µM**			
Wildtype SOD1 ***	75.5±0.1	56.0±0.0	8.8×10^−6^
SOD1^G93A^ **	68.7±0.1	54.1±0.0	1.6×10^−5^
GFP-SOD1^G93A^ **	81.2±0.0	83.0±0.2	4.1×10^−3^
**11µM**			
Wildtype SOD1 **	73.8±0.04	54.2±0.1	1.7×10^−5^
SOD1^G93A^ ***	67.8±0.06	52.7±0.0	5.3×10^−6^
GFP-SOD1^G93A^ ***	79.2±0.00	80.6±0.0	5.6×10^−6^

The thermal stability of wildtype SOD1 and SOD1^G93A^ mutants was assessed at two concentrations (11µM and 33µM) and in the metallated and demetallated form (when incubated with 20mM EDTA which functioned as a heavy metal chelator). Protein stability is expressed as apparent Tm, which refers to the mid-point temperature (°C) where the ratio of unfolded to folded proteins is 1∶1. Apparent Tm values reported are the average values±SD obtained from n = 3. Asterisks denote p-values calculated using the two-tailed Student t-test for the paired condition of metallated versus demetallated SOD1, where ** = p<0.005 and *** = p<10^−5^.

### Assessing Tertiary Structure and Loss of Fibrillization Property of SOD1 when Fused with GFP

**Figure 2:**
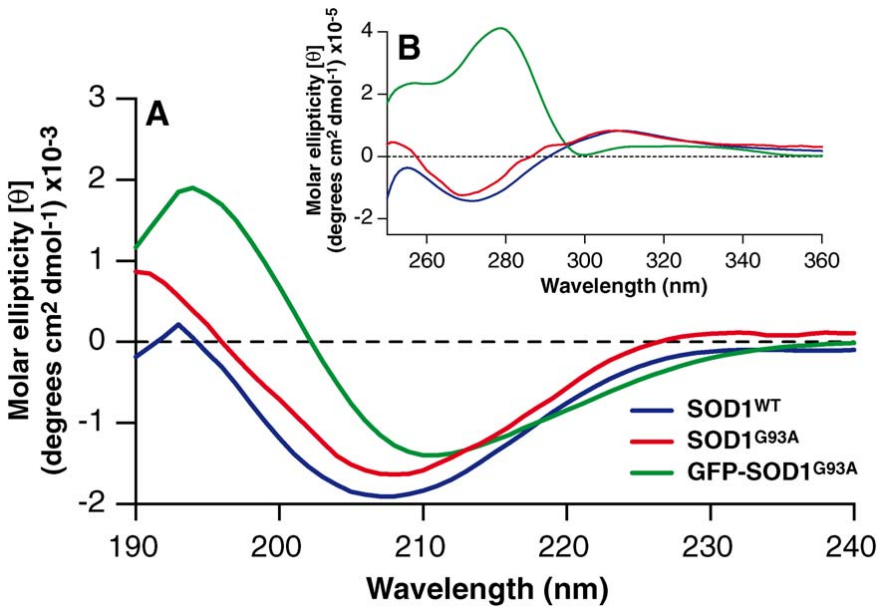
Circular dichroism (CD) spectra of SOD1 proteins. (A) Far-UV CD spectra at wavelength range of 190–240 nm. Wildtype SOD1 (blue) and SOD1^G93A^ (red) shared similar CD spectra, which closely resembled spectra of proteins containing β-sheet conformation. The CD spectra of GFP-SOD1^G93A^ (green), although slightly different from spectra of non-tagged SOD1 proteins, also resembled spectra of proteins containing a β-sheet conformation. (B) Near-UV CD spectra at wavelength range of 250–360 nm. The near-UV CD spectra of non-tagged SOD1 proteins were similar to each other suggesting a preserved tertiary structure of SOD1 regardless of the presence of mutation.

To determine if the fusion of GFP caused any gross alterations in the structure of SOD1, purified proteins were characterized by circular dichroism to compare the secondary (far-UV) and tertiary (near-UV) structures. As shown in Figure 2 the secondary structures of SOD1^WT^ and SOD1^G93A^ both shared a minimum peak at ∼208nm. These spectra showed slight deviation of 3nm from the normal minimum peak for beta-strand structures, at 211nm. This deviation was not investigated further as it was considered insignificant and the protein purified here was comparable to SOD1 proteins reported by others as having a similar peak deviation [13], [14], [15]. In contrast to the far-UV spectra of GFP-SOD1, the minimum peak was at approximately 211nm, which was similar to that for proteins rich in beta-strand structures. The stronger spectral signal at 211nm for GFP-SOD1^G93A^, compared to non-GFP tagged SOD1, may have been due to the presence of an additional 11-beta strands in the GFP moiety, giving a total of 19 beta strands in GFP-SOD1 per monomer. It was unlikely that the beta-strand signal in the far-UV spectrum was due to dimeric GFP because, as in Figure 1, GFP-SOD1^G93A^ mainly existed as GFP-SOD1^G93A^ dimers, and not as monomers, or dissociated GFP-SOD1^G93A^ with GFP forming homodimers. Based on these qualitative assessments, we conclude that there is no significant difference in the secondary structure of untagged and GFP-tagged SOD1 when assessed in the far UV (190–250nm) spectral region.

The tertiary organisation of GFP-SOD1^G93A^ was significantly different from untagged SOD1^WT^ and SOD1^G93A^. GFP is approximately 1.7 times larger in size than a 16kDa monomeric SOD1 protein and as the GFP-tagged SOD1 was shown by AUC to exist in a dimeric form, this means each dimeric SOD1 has two GFP tags likely localized in the proximity of the SOD1 dimer interface. This probably explains why GFP-SOD1^G93A^ has significant overall tertiary structural changes compared to the untagged SOD1 and this difference should not be overlooked when considering toxic properties, including possible fibrillisation properties, of mutant and wildtype SOD1 compared to the untagged forms.

**Figure 3:**
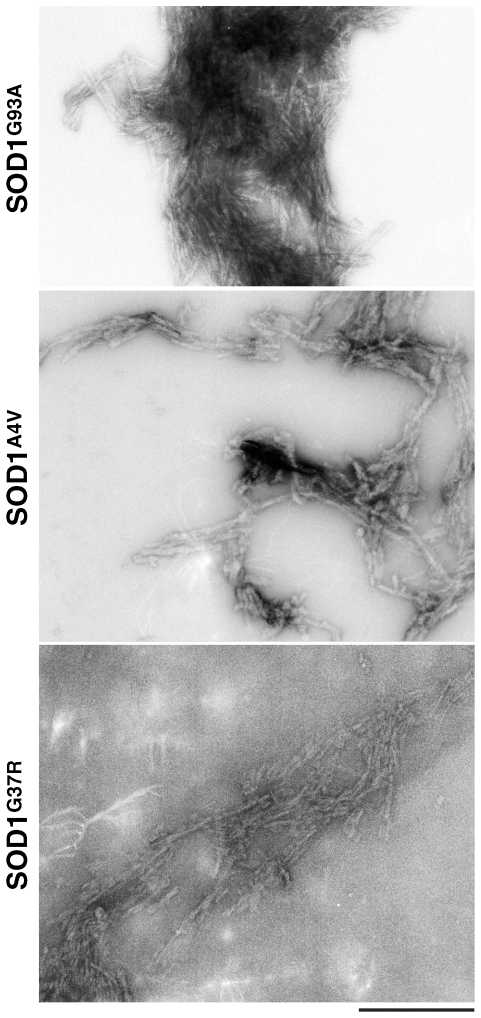
Representative electron micrographs of amyloid-like structures of mutant SOD1 from *in vitro* fibrillization assays. Fibrillization of SOD1 variants SOD1^G93A^, SOD1^A4V^, SOD1^G37R^ was carried out at pH 4 with 0.5 M GdnHCl. All SOD1 fibril samples showed an increased propensity to clump together or to aggregate, regardless of the morphology of the ThT-positive species (scale bar = 400 nm).

Consistent with previous reports suggesting the role of fibrillization or oligomerization of SOD1 in the pathogenesis of ALS [14], [16], we were able to demonstrate the ability of SOD1^G93A^ to form amyloid-like structures under some destabilizing conditions, *in vitro*. To assess the specificity of SOD1^G93A^ fibrillization six other SOD1 mutants (SOD1^A4V^, SOD1^G37R^, SOD1^D83G^, SOD1^G85R^, SOD1^D90A^ and SOD1^G114A^) were confirmed to form fibrils under similar destabilizing conditions as SOD1^G93A^ [17], [18]. The presence of SOD1 amyloid-like species was confirmed by electron microscopy, as shown in Figure 3 [17]. In contrast to SOD1^WT^ and SOD1^G93A^ (and the six other SOD1 mutants), GFP-SOD1^G93A^ failed to oligomerize under conditions which were conducive for SOD1^G93A^ fibrillization. These results show that although some protein properties of GFP-SOD1^G93A^ were similar to SOD1^WT^ and SOD1^G93A^, a possible toxic property of SOD1, the ability to form oligomers, was impaired.

### Protein Expression and Cellular Localization of SOD1 Variants in PC12 Cells

**Figure 4:**
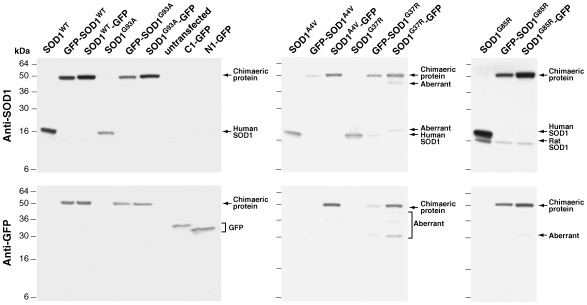
Western blots of PC12 cell lysates from cells transfected with wildtype or mutant SOD1, untagged or GFP tagged at the amino and carboxy termini of SOD1, probed with anti-human SOD1 and with anti-GFP. All proteins are shown in the order: untagged (e.g. SOD1^WT^), GFP-tagged at the amino-terminus of SOD1 (e.g. GFP-SOD1^WT^), GFP-tagged at the carboxy-terminus of SOD1 (e.g. SOD1^WT^-GFP). Lysate from PC12 cells transfected with pAcGFP1-C1, N1-GFP similarly is derived from cells transfected with pAcGFP1-N1, both are controls for GFP alone. Anti-SOD1 antibodies used are: NCL-SOD1 (Novocastra) for G85R mutants, SC-17767 (Santa Cruz) for all other forms of SOD1.

PC12 cells were transfected with constructs encoding SOD1^WT^ or the mutants SOD1^G93A^, SOD1^A4V^, SOD1^G37R^, SOD1^G85R^, the untagged form or tagged at either the N- or C-terminus with GFP. After transfection cell lysates were western blotted and probed with antibodies against human SOD1 and against GFP (Figure 4). In all cases, bands were detected at the expected mass of the chimaeric protein (43kDa) (or that of the individual proteins in control lanes (16kDa, SOD1; 27kDa, GFP). However, lower molecular weight bands (‘Aberrant Bands’, Figure 4) were detected from both N- and C-tagged SOD1^G37R^ transfected cells, suggesting that either a truncated protein had been synthesised or partial degradation had taken place, perhaps generating separate SOD1 and GFP proteins as these bands had sizes of 27 and 16kDa; at least some complete chimaeric SOD1^G37R^ protein was produced. There were also faint aberrant bands at 18 kDa and 36 kDa in the anti-GFP lane of SOD1^A4V^-GFP and at 27kDa in the anti-GFP lane of SOD1^G85R^-GFP. This suggests that similar, but less prominent processes are occurring to these mutants, both of which are tagged with GFP at the C-terminus of SOD1.

**Figure 5:**
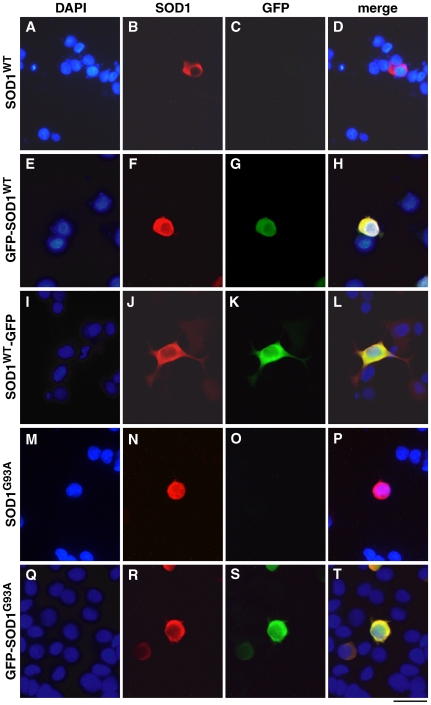
Intracellular distribution of SOD1^WT^, GFP-SOD1^WT^, SOD1^WT^-GFP, SOD1^G93A^, GFP-SOD1^G93A^ in transfected PC12 cells. (A–D) wildtype untagged SOD1 (A) DAPI stain (blue), (B) anti SOD1 (red) using sc-17767 primary antibody, (C) GFP, (D) merged image; (E–H) GFP-SOD1^WT^; (I–L) SOD1^WT^-GFP; (M–P) SOD1^G93A^; (Q–T) GFP-SOD1^G93A^. Scale bar: 20µm.

To compare the cellular localisation of tagged and untagged SOD1, PC12 cells transfected with SOD1^WT^, GFP-SOD1^WT^, SOD1^WT^-GFP, SOD1^G93A^ and GFP-SOD1^G93A^ constructs were immunostained with an antibody against human SOD1. For each construct, a minimum of 100 cells was observed and no discernable difference was detected in localisation of SOD1 (Figure 5).

### Assessing GFP-SOD1 Localisation in Primary Cultured Spinal Motor Neurons

**Figure 6:**
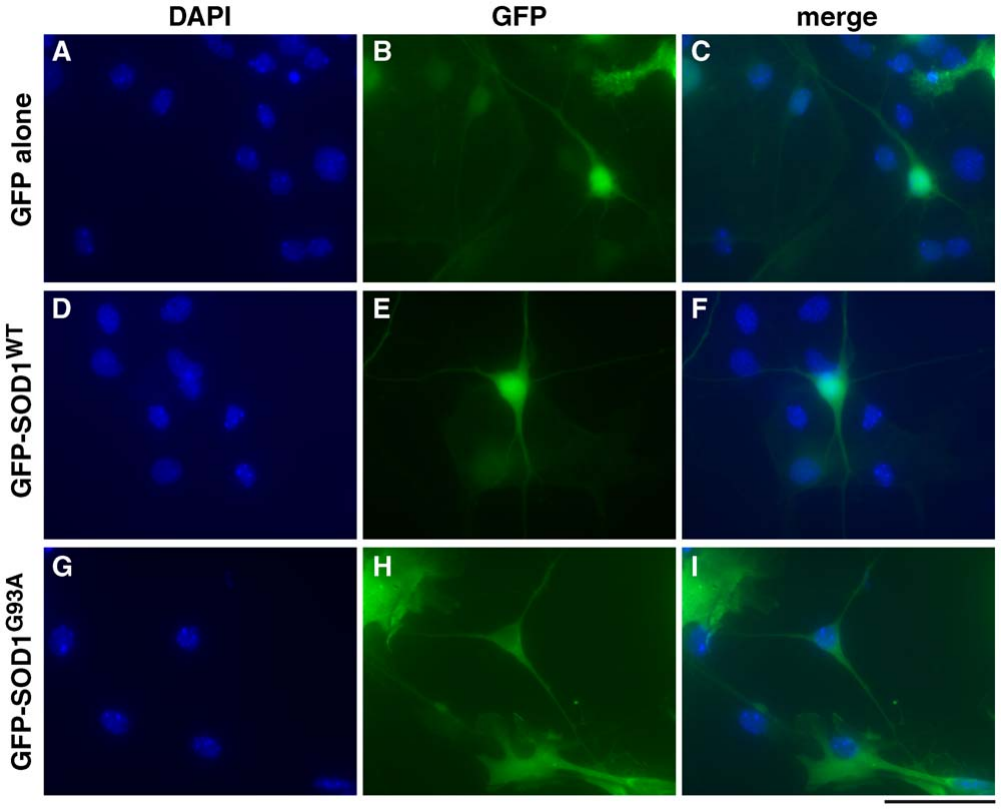
Intracellular distribution of GFP-SOD1^WT^, GFP-SOD1^G93A^ in embryonic spinal motor neurons. Cultured E13.5 wildtype spinal motor neurons were transduced using vectors expressing (1) GFP alone, (2) GFP-SOD1^WT^ (GFP at the amino-terminus of wildtype SOD1), and (3) GFP-SOD1^G93A^ (GFP at the amino-terminus of mutant SOD1). (A–C) GFP alone: (A) DAPI stain, (B) GFP, (C) merged. (D–F) GFP-SOD1^WT^: (D) DAPI stain, (E) GFP, (F) merged. (G–I) GFP-SOD1^G93A^: (G) DAPI stain, (H) GFP, (I) merged. Scale bar: 50 µm. Non-neuronal cells which form part of the spinal cord cultures are also visible in these images. All cells show nuclear DAPI staining and the cytoplasm of non-neuronal cells which have been transduced by lentivirus is visible as irregularly-edged GFP fluorescence.

To compare the behaviour of GFP and GFP-tagged SOD1, three inserts were subcloned into a lentiviral vector: (1) GFP alone, (2) GFP-SOD1^WT^ (GFP at the amino-terminus of wildtype SOD1), and (3) GFP-SOD1^G93A^ (GFP at the amino-terminus of mutant SOD1). Cultured primary mouse embryonic spinal motor neurons were transduced with recombinant lentivirus, cultured for 5 days and fixed and counted. The intracellular distribution of GFP, GFP-SOD1^WT^ and GFP-SOD1^G93A^ was compared and none of these proteins showed a statistically significant tendency to form focal accumulations. The predominant distribution observed was uniform, throughout the cytoplasm, axon and dendrites and no differences were detectable (Figure 6).

**Figure 7:**
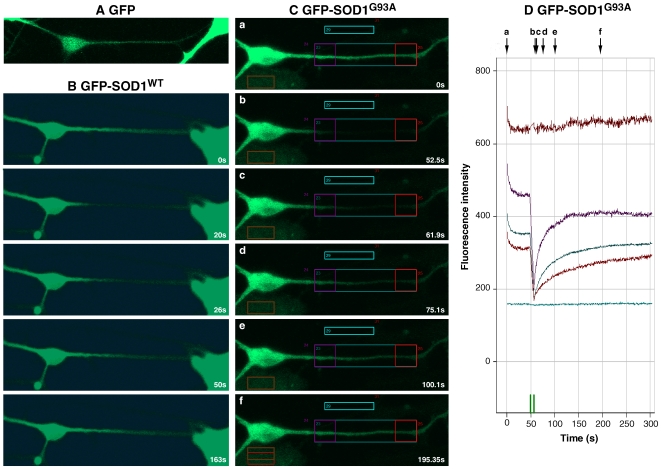
Fluorescence recovery after photobleaching (FRAP) in transduced mouse embryonic spinal motor neurons. (A) motor neuron expressing GFP alone prior to photobleaching. (B) motor neuron expression GFP-SOD1^WT^. Before photobleaching (0s) and after photobleaching (times shown in lower right corner). (C) Motor neuron expressing GFP-SOD1^G93A^. Before photobleaching (0s) and after photobleaching (times shown in lower right corner). (D) Quantification of FRAP from (C). The regions of interest (ROIs) are shown as coloured boxes in images a–f. The intensity of fluorescence from these individual ROIs over time is shown (D). The colour of the individual boxes (ROIs) on images a–f corresponds to the colour of the line plotted on graph (D) for that ROI. Note how the exponential recovery seen on the curves of the graph corresponds to the movement of GFP back into the bleached axon over time. The time course of the experiment is shown in the bottom right corner of images a–f as well as on the X-axis of the graph (D). The time points at which each image a-f was taken are shown along the upper margin.

The dynamics of these chimaeric proteins were also observed using live-cell confocal video microscopy, which necessitates using GFP tagged proteins only. All neurons showed uniform cytoplasmic distribution without evidence of accumulation in discrete foci or of individually transported quanta. To assess movement of GFP, GFP-SOD1^WT^ and GFP-SOD1^G93A^ further, sections of axon from wildtype motor neurons were studied using Fluorescence Recovery After Photobleaching (FRAP) and the FRAP recovery curves recorded. Kinetic Image analysis software was used to fit a halftime of recovery to the FRAP curves and to calculate mobile fractions. Bleached areas of axon showed no evidence of quantal transport and fluorescence recovered following diffusion kinetics (Figure 7, and example of GFP- SOD1^G93A^ shown in Movie S1).

Table 2 shows the mobile fractions and the halftimes (T_1/2_) of the neurons studied in this experiment. No statistically significant difference was found between movement rates of GFP, GFP-SOD1^WT^ and GFP-SOD1^G93A^ or of the mobile and immobile fractions present within the bleached areas.

**Table 2:** Mobile fractions and halftimes from FRAP analysis of GFP, GFP-SOD1^WT^ or GFP-SOD1^G93A^ in WT motor neurons.

Protein	Neuron	Mobile Fraction	T_1/2_/s
GFP	1	0.728	42.771
	2	0.700	55.261
	3	0.675	38.764
	4	0.746	21.241
	5	0.736	30.406
GFP-SOD1^WT^	1	0.801	118.867
	2	0.860	35.179
	3	0.777	44.538
	4	0.736	53.125
	5	0.900	22.487
GFP-SOD1^G93A^	1	0.866	16.745
	2	0.941	30.460
	3	0.729	172.867
	4	0.728	57.640
	5	0.725	81.506

15 wildtype motor neurons expressing either GFP, GFP-SOD1^WT^ or GFP-SOD1^G93A^ were studied using FRAP and the FRAP recovery curves recorded. Kinetic Image analysis software was used to fit a halftime of recovery to the FRAP curves and to calculate a mobile fraction.The halftime values of the main 50µm ROI were plotted on a Q-Q plot – comparing observed and expected valueswhich demonstrated the data were not distributed normally.. To test whether there was a difference between halftime values of wildtype neurons expressing GFP, GFP-SOD1^WT^ or GFP-SOD1^G93A^, a Kruskal-Wallis test was applied. This test is a non-parametric means of comparing groups of results. A significance value of less than 0.05 implies a statistically significant difference between the groups studied. The results of this analysis are for the Mobile fraction: Chi-square value = 5.180; Degrees of Freedom = 2; Significance level = 0.075. As the significance level is greater than 0.05, this demonstrates that the mobile fractions of GFP, GFP-SOD1^WT^ or GFP-SOD1^G93A^ have not been shown to be different under the test conditions used.

## Discussion

Here, we report on the biophysical and biochemical effects of tagging human SOD1 protein with GFP, by comparing untagged and tagged wildtype and mutant SOD1. We found the aromatic CD spectrum was significantly different when compared between untagged and GFP-tagged SOD1 proteins and likely dominated by the contribution of GFP. Although GFP-SOD1^G93A^ exists as a stable dimer as shown by AUC, metallated GFP-SOD1^G93A^ differed in its specific enzymatic activity by only having 35% activity compared to untagged SOD1^WT^ and SOD1^G93A^. Furthermore, metallated GFP-SOD1^G93A^ also had an altered apparent thermal stability compared to the untagged metallated SOD1^WT^. In addition, for undetermined reasons, we found loss of Cu/Zn ions from GFP-SOD1^G93A^ unexpectedly caused an increase in stability which was reflected as an increment in the apparent Tm which, when compared to untagged SOD1^WT^ is likely due to additional stability contributed by the GFP-moiety. We note that the changes we detect in vitro on thermal stability may not reflect what happens within a cell.

Unexpectedly, we also found that GFP-SOD1^G93A^ was unable to form fibrils in conditions where untagged forms are efficient at fibril formation [17], [18]. This is a potentially important difference for current studies of mutant SOD1 pathogenesis. Nevertheless, most SOD1 fusion proteins were stable when expressed in PC12 cells, although a subset were cleaved; we note that under experimental conditions to detect GFP, free GFP or truncated GFP-SOD1 fusion proteins may be detected, rather than intact SOD1-GFP fusion proteins.

The indistinguishable cytoplasmic distribution of GFP alone, GFP-SOD1^WT^ and GFP-SOD1^G93A^ in PC12 cells and cultured embryonic motor neurons and the indistinguishable FRAP characteristics may be a true reflection of the solubility and natural distribution of SOD1^WT^ and SOD1^G93A^. However, we note that we do not have high resolution confocal microscopy images of the intracellular protein distribution from these constructs. Durham et al [19] reports that untagged mutant SOD1 forms accumulations within the cytoplasm whereas untagged wildtype SOD1 does not when expressed in cultured motor neurons; Takeuchi and co-authors, and Matsomoto and co-authors report similar results in other cell systems [6], [8]. Clearly the intracellular aggregation of tagged and untagged SOD1 in motor neurons remains to be examined.

The value of GFP as a protein tagging tool has been proven on multiple occasions and is beyond doubt. Comparing mutant and wildtype tagged proteins in transfected cells is a well-established experimental paradigm. Our results show that a detailed study of the biochemical properties of tagged proteins is required to confirm the neutrality of GFP conjugation before an interpretation of subsequent findings is made. When differences are seen between the behaviour of mutant and wildtype tagged proteins, these should not be taken as unequivocal representations of the behaviour of these proteins in the untagged state but rather as pointers towards them, to be confirmed by multiple interacting lines of enquiry.

## Materials and Methods

### Ethics Statement

All work involving mice was carried out under Licence from and in accordance with UK Home Office requirements.

### Expression and Purification of Human SOD1 Variants

The open reading frame (ORF) sequence of the wildtype *SOD1* cDNA was confirmed by sequencing pSP64 [20] and comparing the *SOD1* open reading frame to File Accession no.: NM_000454 from the Nucleotide database on NCBI (http://www.ncbi.nlm.nih.gov). Using pSP64 template, site directed mutagenesis was performed to generate constructs for SOD1^G93A^ and the other mutations described. GFP-tagging was achieved by subcloning SOD1 cDNAs into the vector pAcGFP1-C1 (Clontech) between the *EcoRI* and *SalI* restriction sites. Constructs were cloned into the pET28 expression vector (Novagen), downstream of the T7 promoter between *NdeI* and *XhoI* sites introducing a thrombin cleavage site between the protein and the N-terminal His-tag. All constructs were transformed into *Escherichia coli* strain BL21 (DE3) (Novagen) according to manufacturer's protocol. SOD1 expression was induced with 0.1M IPTG at 18^o^C overnight. Recombinant proteins were purified to homogeneity using metal chelate affinity (Ni-NTA, Qiagen) chromatography and the His-tag cleaved with thrombin (Novagen). All chromatography steps were performed at room temperature using a Bio-Rad Bio-Logic chromatography system. Purified SOD1 was metal loaded by dialyzing it against 100 mM Tris-Cl pH8.0, 300 mM NaCl containing 150–200 uM CuSO_4_ over 3–4 hours at room temperature, then followed by ZnSO_4_ and final dialysis into 20 mM Tris-Cl pH 7.5 for storage.

### Lentiviral Constructs

For generation of lentiviral vectors containing the SOD1 mutant variants fused to AcGFP, we used the lentiviral backbone pRRL156sin-cPPT-hCMV-wpre, containing a multiple cloning site. SOD1-AcGFP fragments were cut from the pAcGFP1-C1 plasmid with *NheI* and *SalI* restriction sites. These fragments were then inserted between the *XbaI* and *SalI* sites of the multiple cloning site in pRRL156sin-cPPT-hCMV-wpre. To produce viral vectors, the LV-SOD1AcGFP transfer plasmid was co-transfected with the viral core packaging construct pCMVdeltaR8.74 and the VSV-G envelope protein vector pMD.G.2 into 293T cells as previously described [21]. Briefly, 5×10^6^ 293T cells were seeded in 10 cm dishes 24 hrs prior to transfection in complete Iscove's Modified Dulbecco's Medium (IMDM) containing 10% FCS, 1× glutamine (Gln) and 1× penicillin/streptomycin (PS) and grown in a 5% CO_2_ incubator. Two hrs prior to transfection the culture medium was changed. For each 10 cm dish 10 µg of transfer vector plasmid, 6.5 µg envelope plasmid and 3.5 µg of packaging plasmid were co-transfected using the calcium phosphate method. The medium was replaced with IMDM containing 2% FCS, 1×Gln and 1×PS after 14–16 hrs and LV particle containing medium was collected 24 hrs later, cleared by low-speed centrifugation (176×g for 5 minutes) and filtered through a 0.22 µm cellulose acetate filter. The supernatant was concentrated about 100 fold by ultra centrifugation (53,000×g for 2.5 hrs). The pellet was re-suspended in PBS and aliquots of LV-SOD1AcGFP stored at −80°C. The titer (transducing units/ml; TU/ml) was determined by transducing 293T cells with serial dilutions of the LV-SOD1AcGFP vector in complete IMDM. Transduced cells were incubated for 24 hrs and thereafter medium was replaced with fresh IMDM and cells were incubated for another 24 hrs. The titer was determined by counting GFP positive cells under a fluorescence microscope and was 1.5×10^9^ TU/ml.

### Structural, Enzymatic and Stability Characterization

#### Spectroscopic measurements – Circular Dichroism at far and near UV

Circular dichroism (CD) spectroscopy was performed using a Jasco J-715 spectrophotometer (Jasco). The secondary and tertiary structure of purified SOD1 was determined by obtaining CD spectra at far-UV (250–190nm) and near-UV (350–250nm) respectively. Far-UV CD spectra were obtained in 0.1 mm path length circular cuvettes, near-UV spectra were sampled in 10 mm path length standard quartz cuvettes. All data were collected using a stop resolution of 1 nm, a scan speed of 50 nm/min, a 1 second response time. Measurements were performed over 10 accumulations to reduce signal to noise ratio and baseline corrected against storage buffer. Protein concentrations were approximately 1.0–1.5 mg/ml, and CD measurements were converted to units of molar ellipticity ([θ]). All corrections and processing were undertaken using the Jasco Standard Analysis Program.

### Measurement of SOD1 Enzymatic Activity

SOD1 activity was determined at relevant steps throughout purification using a commercially available kit, Bioxytech SOD-525 assay (Oxis Research) according to manufacturer's protocol. Activities for SOD1 variants are expressed as % activity of wildtype human SOD1.

### Differential Scanning Fluorimetry

Thermal unfolding of SOD1 variants was monitored using SYPRO Orange and was performed by using a real-time PCR instrument (Applied Biosystem TaqMan RT-PCR). Experimental design was adapted from the published protocol of [22]. Briefly, using a 96-well PCR plate, SYPRO Orange was mixed with protein in 1∶1000 ratio with final volume of 20µl/well (triplicates/sample and with/without EDTA at 11µM and 33µM) and sealed with an Optical PCR seal. An RT-PCR instrument was programmed to run with increasing temperature gradient from 19°C–95°C with 1°C increment and to hold for 60 sec at every new temperature. Fluorescence was monitored using the in-built filters: FAM (492 nm) and ROX (610 nm), for excitation and emission, respectively. The fluorescence intensity is plotted as a function of temperature and curve-fitted according to the Vant Hoff equation to determine the apparent Tm value (temperature at mid-point of unfolding) using GraFit software.

### Analytical Ultracentrifugation (AUC)

Wildtype SOD1, SOD1^G93A^ and GFP-SOD1^G93A^ were analysed by sedimentation velocity experiments using a Beckman Optima XL-I (Beckman Coulter). 400µl protein was loaded into a double sector Epon cell with sapphire windows and centrifuged at 50,000rpm (182 000rcf at cell centre and 201 600rcf at cell bottom) at 20°C using an An-50Ti rotor (Beckman Coulter). Samples were monitored immediately on reaching maximum speed using interference optics and then a further 199 scans were taken every 5 mins. Every other scan was analysed using Sedfit Version 9.2, (20) using the continuous size distribution (c(s)) method entering no prior knowledge. Values for the partial specific volume of the protein and density and viscosity of the buffer used were calculated using Sednterp, [23] and entered into the model parameters. This gave a distribution in sedimentation coefficients which was then converted to molar mass using the molar mass distribution (c(M)) model. c(s) units are fringes per Svedberg, c(M) units are fringes/Da. 1 fringe is approximately 0.3mg/ml.

### *In Vitro* Conversion of SOD1 into Fibrils

To form amyloid fibrils a stock solution of SOD1 protein, wildtype and mutants (SOD1^G93A^ and GFP-SOD1^G93A^) (1.3–1.5 mg/mL) was diluted to the final protein concentration of 10 µM in 20 mM Tris-acetate buffer, with varying concentrations of guanidinium HCl and at pH 4.0, 5.0, 7.5 and/or 9.0), and final 10 µM Thioflavin-T (ThT). Four Hybaid Ribolyser beads were placed into each well of a 96-well transparent flat-bottom plate; then 200 µl reaction mixture containing diluted protein and ThT was pipetted into wells, and the plates were covered using an optically corrected adhesive film. Assay was incubated at 37°C upon continuous shaking at 830 rpm using a plate incubator shaker (GrantBio). The kinetics of fibril formation were monitored by taking time point measurements of fluorescence emission at 485 nm when excited at 450 nm using a fluorimeter plate reader (Spectrofluor, Tecan). For seeded reactions, 2 µl of preformed fibrils from spontaneous reaction were added to 200µl reaction as described above.

The lag phase of amyloid formation was determined by fitting the time-dependent changes in the fluorescence of ThT (*F*) over time of the reaction (*t*) to the following equation:
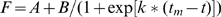
where *A* is the base level of ThT fluorescence during the lag phase, *B* is the difference between final level of ThT fluorescence at plateau and the initial level during the lag phase, *k* is the rate constant of fibril growth (h^–1^), and *t_m_* is the observed time at midpoint of transition. The lag time (*t_l_*) of fibril formation was calculated as: *t_l_* = *t_m_*–2/*k*.

### Electron Microscopy

Samples were prepared for negative staining on carbon grids by applying 3.5µl of sample (fibrils or native protein) to a glow discharged, carbon-coated 300-mesh copper grid and blotted after 2–3mins. The grids were stained with 3.5µl 2% (w/v) uranyl acetate, blotted after 2mins and allowed to air-dry. Images were recorded using minimal electron dose at a magnification of 27 000× in a Tecnai T10 microscope (FEI, Eindhoven, NL) with a tungsten filament operating at 100kV. Grid preparation, analysis and imaging were performed by Dr. Howard Tattum (MRC Prion Unit, UK).

### Transfection of PC12 Cells and Western Blotting

PC12 cells were seeded at 24,000 cells/cm^2^ in 10cm diameter Petri dishes. These were transfected using Lipofectamine 2000™ according to manufacturer's instructions. The following constructs were used: *SOD1 untagged*: (1) pcDNA3.1(+)SOD1^WT^, (2) pcDNA3.1(+)SOD1^G37R^ (3) pcDNA3.1(+)SOD1^A4V^, (4), pcDNA3.1(+)SOD1^G85R^, (5) pcDNA3.1(+)SOD1^G93A^; *GFP tagged at the SOD1 amino terminus*: (6) pAcGFP1-SOD1^WT^, (7) pAcGFP1-SOD1^G37R^, (8) pAcGFP1-SOD1^A4V^, (9) pAcGFP1-SOD1^G85R^, (10) pAcGFP1-SOD1^G93A^; *GFP tagged at the SOD1 carboxy terminus*: (11) pSOD1^WT^-AcGFP1, (12) pSOD1^G37R^-AcGFP1, (13) pSOD1^A4V^-AcGFP1, (14) pSOD1^G85R^-AcGFP1, (15) pSOD1^G93A^-AcGFP1.

At 48–72 hours later (when GFP expression, assessed by fluorescence microscopy, appeared maximal) the culture medium was removed from each dish and 1 ml western blotting cell lysis buffer was added to each dish. Cells were then scraped and cell lysates were collected in microcentrifuge tubes. Total protein concentration of the lysates was assessed by a BCA protein assay kit (Pearce) according to the manufacturer's protocol. For western blotting, 1000 µl 2× SDS sample buffer was warmed to 37°C and added to 400 µl β-mercaptoethanol and 80 µl AEBSF. 60 µl SDS sample buffer/β-mercaptoethanol/AEBSF was added to 60 µl of each cell lysate and heated at 100°C for 10 minutes. Samples (20 µl) were analysed on 16% tris-glycine 1.5 mm pre-cast SDS PAGE gels (1.5m thickness, Invitrogen) at 200V for 70 minutes. Proteins were either stained with Coomassie blue or blotted on to Immobilon P membrane for 35V for 90 minutes. Immobilon membranes were then blocked using 5% w/v powdered milk in PBST for 1 hour at room temperature. Membranes were washed 3 times in PBST and incubated either with anti-SOD1 (1∶500 solution NCL-SOD1, Novocastra for G85R mutants, SC-17767, Santa Cruz for all other forms) in PBSTor in a 10µg/10ml (1∶500) solution of anti-GFP (Zymed 33–2600) in PBST at room temperature overnight. Membranes were then washed in PBST for 60 minutes, and then incubated with goat anti-mouse secondary (Sigma A2179, 1∶10,000 in PBST) conjugated to Alkaline Phosphatase for 1 hour then were washed in PBST for an hour, changing the PBST every 15 minutes. Membranes were washed twice, five minutes each in 1× Tropix assay buffer, and were incubated with CDP-Star for 5 minutes. Membranes were exposed to BioMax MR-1 film and were developed using an Xograph compact ×4 developer.

### Culture of Motor Neurons

Mixed ventral horn motor neuron cultures were prepared according to a modified version of that described by Arce et al [24]. Timed matings of mice were set up and embryos harvested at day E13.5. Cells were plated at the required density in complete neurobasal medium which was replaced every 48–72 hours.

#### Viral Transduction of Motor Neuron Cultures

A multiplicity of infection of 50 was routinely used for transduction of motor neuron cultures. The volume of viral concentrate required for this volume was calculated and this mixed with the requisite volume of complete neurobasal medium. This mixture was used to replace existing culture medium 24h after neurons had been plated. 24h after medium containing viral concentrate was added, it was replaced by fresh complete neurobasal medium, without viral concentrate.

#### Confocal Microscopy of Live Cultured Motor Neurons

Confocal microscopy was carried out on a Zeiss LSM 510 Meta microscope equipped with an environmental chamber kept at 37°C, using a plan apochromat ×63 NA 1.4 objective. Imaging buffer was warmed to 37°C. All neurons used for live cell imaging were cultured in Mattek dishes. Complete neurobasal medium was replaced with imaging buffer (E4 without phenol red supplemented with 30 mm HEPES–NaOH, pH 7.4).

#### Fluorescence Recovery after Photobleaching (FRAP) Analysis

Neurons were scanned keeping the cell body to the left by convention. Neurons expressing a fluorescent protein with straight, clearly identifiable axons were chosen for FRAP analysis. A segment of axon 50 µM in length was defined as the primary region of interest (ROI 1) using the Zeiss LSM 510 software. Further regions of interest were the proximal (ROI 2) and distal (ROI 3) portions of ROI 1 (both 10µm in length) and fluorescent (ROI 4) and background (ROI 5) reference regions. The neuron of interest was imaged for at least 10 frames before bleaching for 50 iterations at 100% laser power throughout ROI 1. The fluorescence of each ROI was recorded during this time and during fluorescence recovery and FRAP curves of fluorescence vs time recorded.

### Supporting Information

Movie S1 Fluorescence recovery after photobleaching (FRAP) in a transduced mouse embryonic spinal motor neuron. A motor neuron expressing GFP-SOD1^G93A^. Before photobleaching and after photobleaching (time in seconds as shown).
(1.49 MB MOV)

## References

[1] Deng HX, Hentati A, Tainer JA, Iqbal Z, Cayabyab A (1993). Amyotrophic lateral sclerosis and structural defects in Cu,Zn superoxide dismutase.. Science.

[2] Rosen DR, Siddique T, Patterson D, Figlewicz DA, Sapp P (1993). Mutations in Cu/Zn superoxide dismutase gene are associated with familial amyotrophic lateral sclerosis.. Nature.

[3] Gros-Louis F, Gaspar C, Rouleau GA (2006). Genetics of familial and sporadic amyotrophic lateral sclerosis.. Biochim Biophys Acta.

[4] Crapo JD, Oury T, Rabouille C, Slot JW, Chang LY (1992). Copper,zinc superoxide dismutase is primarily a cytosolic protein in human cells.. Proc Natl Acad Sci U S A.

[5] Pardo CA, Xu Z, Borchelt DR, Price DL, Sisodia SS (1995). Superoxide dismutase is an abundant component in cell bodies, dendrites, and axons of motor neurons and in a subset of other neurons.. Proc Natl Acad Sci U S A.

[6] Takeuchi H, Kobayashi Y, Ishigaki S, Doyu M, Sobue G (2002). Mitochondrial localization of mutant superoxide dismutase 1 triggers caspase-dependent cell death in a cellular model of familial amyotrophic lateral sclerosis.. J Biol Chem.

[7] Corti S, Locatelli F, Donadoni C, Guglieri M, Papadimitriou D (2004). Wild-type bone marrow cells ameliorate the phenotype of SOD1-G93A ALS mice and contribute to CNS, heart and skeletal muscle tissues.. Brain.

[8] Matsumoto G, Stojanovic A, Holmberg CI, Kim S, Morimoto RI (2005). Structural properties and neuronal toxicity of amyotrophic lateral sclerosis-associated Cu/Zn superoxide dismutase 1 aggregates.. J Cell Biol.

[9] Matsumoto G, Kim S, Morimoto RI (2006). Huntingtin and mutant SOD1 form aggregate structures with distinct molecular properties in human cells.. J Biol Chem.

[10] Zhang F, Zhu H (2006). Intracellular conformational alterations of mutant SOD1 and the implications for fALS-associated SOD1 mutant induced motor neuron cell death.. Biochim Biophys Acta.

[11] De Vos KJ, Chapman AL, Tennant ME, Manser C, Tudor EL (2007). Familial amyotrophic lateral sclerosis-linked SOD1 mutants perturb fast axonal transport to reduce axonal mitochondria content.. Hum Mol Genet.

[12] Gurney ME, Pu H, Chiu AY, Dal Canto MC, Polchow CY (1994). Motor neuron degeneration in mice that express a human Cu,Zn superoxide dismutase mutation.. Science.

[13] Arnesano F, Banci L, Bertini I, Martinelli M, Furukawa Y (2004). The unusually stable quaternary structure of human Cu,Zn-superoxide dismutase 1 is controlled by both metal occupancy and disulfide status.. J Biol Chem.

[14] Banci L, Bertini I, Durazo A, Girotto S, Gralla EB (2007). Metal-free superoxide dismutase forms soluble oligomers under physiological conditions: a possible general mechanism for familial ALS.. Proc Natl Acad Sci U S A.

[15] Oztug Durer ZA, Cohlberg JA, Dinh P, Padua S, Ehrenclou K (2009). Loss of metal ions, disulfide reduction and mutations related to familial ALS promote formation of amyloid-like aggregates from superoxide dismutase.. PLoS ONE.

[16] Banci L, Bertini I, Boca M, Girotto S, Martinelli M (2008). SOD1 and amyotrophic lateral sclerosis: mutations and oligomerization.. PLoS ONE.

[17] Chia R (2008). Investigation of molecular pathogenesis of amyotrophic lateral sclerosis and mouse models of neurodegeneration..

[18] Chia R, Tattum MH, Jones S, Collinge J, Fisher EMC (2010). Prion-like properties of superoxide dismutase type 1 in amyotrophic lateral sclerosis.. Submitted.

[19] Durham HD, Roy J, Dong L, Figlewicz DA (1997). Aggregation of mutant Cu/Zn superoxide dismutase proteins in a culture model of ALS.. J Neuropathol Exp Neurol.

[20] Lieman-Hurwitz J, Dafni N, Lavie V, Groner Y (1982). Human cytoplasmic superoxide dismutase cDNA clone: a probe for studying the molecular biology of Down syndrome.. Proc Natl Acad Sci U S A.

[21] Naldini L, Blomer U, Gallay P, Ory D, Mulligan R (1996). In vivo gene delivery and stable transduction of nondividing cells by a lentiviral vector.. Science.

[22] Niesen FH, Berglund H, Vedadi M (2007). The use of differential scanning fluorimetry to detect ligand interactions that promote protein stability.. Nat Protoc.

[23] Hayes DB, Laue TM, Philo J (2003). SEDNTERP (Sedimentiation Utility Software).. Amgen Corp.

[24] Arce V, Garces A, de Bovis B, Filippi P, Henderson C (1999). Cardiotrophin-1 requires LIFRbeta to promote survival of mouse motoneurons purified by a novel technique.. J Neurosci Res.

[25] Lebowitz J, Lewis MS, Schuck P (2002). Modern analytical ultracentrifugation in protein science: a tutorial review.. Protein Sci.

[26] Schuck P (2000). Size-distribution analysis of macromolecules by sedimentation velocity ultracentrifugation and lamm equation modeling.. Biophys J.

